# A Case Study of Quantizing Convolutional Neural Networks for Fast Disease Diagnosis on Portable Medical Devices

**DOI:** 10.3390/s22010219

**Published:** 2021-12-29

**Authors:** Mukhammed Garifulla, Juncheol Shin, Chanho Kim, Won Hwa Kim, Hye Jung Kim, Jaeil Kim, Seokin Hong

**Affiliations:** 1 School of Computer Science Engineering, Kyungpook National University, Daegu 41566, Korea; m_garifulla@knu.ac.kr (M.G.); ewsn1593@knu.ac.kr (J.S.); knight970815@gmail.com (C.K.); threeyears@gmail.com (J.K.); 2 Department of Radiology, Kyungpook National University Chilgok Hospital, Kyungpook National University, Daegu 41404, Korea; greenoaktree9@gmail.com (W.H.K.); mamrad@knu.ac.kr (H.J.K.); 3 Department of Semiconductor Systems Engineering, Sungkyunkwan University, Suwon-si 16419, Korea

**Keywords:** deep neural network, quantization, point-of-care, neural processing unit

## Abstract

Recently, the amount of attention paid towards convolutional neural networks (CNN) in medical image analysis has rapidly increased since they can analyze and classify images faster and more accurately than human abilities. As a result, CNNs are becoming more popular and play a role as a supplementary assistant for healthcare professionals. Using the CNN on portable medical devices can enable a handy and accurate disease diagnosis. Unfortunately, however, the CNNs require high-performance computing resources as they involve a significant amount of computation to process big data. Thus, they are limited to being used on portable medical devices with limited computing resources. This paper discusses the network quantization techniques that reduce the size of CNN models and enable fast CNN inference with an energy-efficient CNN accelerator integrated into recent mobile processors. With extensive experiments, we show that the quantization technique reduces inference time by 97% on the mobile system integrating a CNN acceleration engine.

## 1. Introduction

Most of the medical tests are usually performed at central laboratories equipped with special instruments. The advantages of the centralized laboratory approach are that the test results have higher accuracy due to the equipment quality, professional specialists performing analysis, and well-developed laboratory protocols. However, the centralized laboratory approach has its drawbacks, such as time delays and psychological pressure. For example, when a patient undergoes a diagnosis with healthcare professionals, the patient might have to wait for the results for days, which can lead to the anxiousness of the former or even worse. Moreover, it might cause an aggravation of the illness before it is diagnosed. Thus, centralized laboratory tests are not mobile enough to be used for rapid mass tests.

One of the solutions to decentralize the disease diagnosis process is to use portable medical devices designed for healthcare professionals to perform bedside tests and receive the results immediately. Additionally, the portable medical device can be used by patients themselves while following instructions made by healthcare professionals. For example, mobile devices are being used to simplify and automate analysis in optical microscopy [1]. The ubiquitous mobile phones that we use in daily life can be utilized as a microscope device by attaching printed lenses on smartphone’s camera [2]. Recently, the issue of mobility becomes more apparent if we consider the COVID-19 pandemic, where rapid mass tests are important. The recent research [3] discusses the utilization of a smartphone-based fluorescence microscope device, which is portable and more sensitive than the current RT-PCR (reverse transcription-polymerase chain reaction) gold standard device for diagnosing COVID-19 patients.

The portable medical devices with a deep neural network model will assist professionals to diagnose patients in a short time and carry out required medical treatment [4]. Some recent results demonstrated the use of machine learning models to improve smartphone microscopy imaging [5]. However, running a neural network (NN) model on a computationally limited device or mobile device is difficult. To efficiently run NN models on mobile devices, several parameters, such as latency, accuracy, model size, and energy consumption, need to be improved. The computational demands of NN models can push the devices to their limits. Additionally, computationally heavy processes can significantly stress on the processing units, leading to escalation in energy consumption. Furthermore, deploying the NN model on such equipment causes hardware throttling problems.

Quantization techniques have become popular among other neural network optimization techniques, which have been proposed to overcome the challenges and ease the computational load on mobile devices. Quantization techniques involve the use of low-precision floating-point or integer-format instructions to reduce the inference latency and reduce the size of the deep neural networks model. They can also enable the integer computations to take advantage of hardware accelerators with little degradation in model accuracy.

This paper demonstrates the feasibility of deploying convolutional neural network (CNN) models with post-training quantization to detect benign and malignant breast cancer tumors on portable ultrasound devices. We train the CNN models on ultrasound images of breast cancer patients by using the TensorFlow (TF) framework and converting TF models into TFLite [6] models, which can be run on mobile devices. During the models’ conversion, the post-training quantization [7] takes place. We use various quantization techniques, including dynamic range, half-precision floating-point, and full integer quantizations. In dynamic range quantization, weights are quantized to integer format, and the activation values are quantized and de-quantized before and after multiplication and accumulation processes. The half-precision floating-point quantization technique allows for the quantization of all weights and activation values to the float16 format. Finally, a full-integer quantization converts all weight and activation values into integer format (8-bit integer), which is advantageous due to the lower computation and storage requirements.

This paper analyzes the various quantization techniques for the different CNN models, which are trained with images of breast cancer patients. The quantized CNN models are used to determine the inference accuracy and time on a mobile device. We used the Snapdragon 865 hardware development kit (HDK) [8] as a testing device for our models. The accuracy of the quantized models was evaluated using a Python application on a server-class computing system and an Android application installed on the Snapdragon 865 HDK. In the case of the Snapdragon HDK, the model testings were carried out by utilizing different types of hardware such as CPU, GPU, and NPU (neural processing unit).

The experimental results show that the CNN model’s inference time and accuracy vary depending on the quantization method applied to the model and the hardware used for the inference. Overall, the model quantization can reduce the model size by 75% and the inference time by 97%. We also show that the full-integer quantization has to be applied to the model in order to utilize the NPU (neural processing unit), which is custom hardware designed for the neural network applications. By quantizing the model with the full-integer quantization and using the NPU for the quantized model, the per-image inference time is significantly reduced. These experimental results suggest that the model quantization is essentially applied to the CNN models for rapid patient diagnosis procedures using portable medical devices. In addition, the researchers should employ an appropriate quantization method depending on the hardware type used for the model inference while considering the trade-offs in accuracy, inference time, and model size.

In summary, this paper makes the following contributions:

- We analyze the impact of various quantization techniques on the inference accuracy, model size, and inference time for various CNN models implemented for detecting malignant or benign breast tumors.
- We provide insight about using hardware accelerators widely integrated into the latest mobile processors to accelerate neural network applications on portable medical devices.
- We point out that an appropriate quantization technique should be applied to a model to optimize performance and accuracy trade-offs on specific computing hardware.

The structure of the paper is as follows. Section 2 describes related works in portable medical devices using neural networks and hardware acceleration of neural network models. Section 3 describes the background of CNN and various quantization techniques. In Section 4, we detail the datasets and CNN models used in this study. Then, we analyze the effects of the quantization techniques applied to the CNN models in Section 5. In Section 6, we introduce hardware acceleration types. Section 7 and Section 8 describe the experimental setup and discuss experimental results. Finally, we conclude the research results and outline the importance of model quantization in Section 9.

## 2. Related Work

### 2.1. Medical Image Analysis with Convolutional Neural Networks

The potential of CNN to learn and extract the patterns grants us numerous possibilities to deploy CNN, and the medical field is not out of this scope. As mentioned in previous sections, the medical field is becoming more and more interested in utilization of CNNs since an enormous number of data are generated every day. CNNs offer precious information when it is combined with “traditional” methods of analysis. Furthermore, CNNs are even able to surpass those “traditional” methods. CNNs are used not only as a tool in diagnostics to support clinical decision making but also to predict seizures [9], cardiac arrests [10], cancers [11], and other diseases that require immediate treatment. Tumor segmentation and classification processes are not easy tasks even for CNNs due to the spatial data getting lost in deeper layers and the size of tumor cells that complicate the detection task. This problem was addressed by implementing a feature enhancer [12] that extracts features from shallow layers and adds them to deeper layers. Another example of CNN utilization is brain tumor segmentation. In [13], the authors propose a model that uses a novel cross-modality deep feature learning framework with two learning options: the cross-modality feature transition and cross-modality feature fusion. The problems of computer-aided diagnosis for dermatological diseases are discussed in [14]. The authors addressed the problems of segmentation lesions with uneven forms and unrecognizable boundaries with low contrast by proposing a novel multi-scale residual encoding and decoding network. There is research that concentrates on breast cancer classification using the CNN technique to identify the mitotic and non-mitotic cells [15].

Despite the slow development and integration of neural networks as well as the difficulty in explaining the internal mechanics [16], it is becoming a hot topic worldwide, and some initiatives are moving the research forward. Other non-medical companies use deep neural networks for healthcare projects, such as Microsoft [17], Google Brain [18,19], and IBM [20]. Additionally, the development of deep neural networks and AI can be seen in some other research papers [21].

### 2.2. CNN Quantization

Due to the massive computational requirements of recent CNN models and the ubiquitousness of edge devices, the need for model optimization methods is gaining popularity. One of the methods is known as quantization. There have been a number of publications on deep neural network quantization topics, and NN quantization leaves room for improvement. Low-precision computation allows for more efficient use of hardware capacity, which leads to better efficiency in terms of power consumption [22]. For example, there are research papers related to half-precision as well as mixed-precision training of NN [23,24] in order to achieve acceleration by using general-purpose hardware such as GPU. There are also research papers related to the integer quantization [25,26,27] and low-precision quantization experiments [23]. Most of the quantization methods are uniform quantization type since non-uniform quantization techniques are difficult to deploy on general-purpose hardware [28,29]. Among numerous types of quantization, post-training quantization [29,30,31] stands out due to the simplicity of its implementation, and there is no requirement to retrain the model.

### 2.3. Hardware Acceleration for Quantized CNN

CNN models are becoming more complex, and thus computational requirements are also increasing, leading to higher power consumption. Recently, GPU has been integrated into mobile processors to meet the computational demands of CNN models. Despite GPU integration, the computation requirements push the power consumption to an even higher level, which impacts the power management of mobile devices. Furthermore, the GPU inside of the mobile processor is a type of general-purpose hardware and thus performs redundant computations during CNN model inference time. Therefore, the next step in CNN application on the mobile processor is the development of domain-specific hardware accelerators. That being the case, such hardware shows great performance results while lowering the power consumption rate during model inference. Some hardware accelerators have improved parallel computation and optimized memory hierarchy [32]. Meanwhile, others rely on an array of processing engines (PE) that were introduced in TPU [33] and in other similar types of hardware [34]. The device that contains PEs is mostly referred to as NPU. NPU is designed so that each array of PEs executes the computation of the NN segment, which eases the computational burden. Therefore, PEs handle the weight and activation data of CNN. Moreover, depending on the type of dataflow pattern, they may differ in design such as stationary output dataflow [35], stationary input data flow [36], and stationary weight dataflow [33]. In the case of a combination of quantization and hardware acceleration, both gain an advantage in MAC operations. Since the integer quantization lowers the float bit-width to a fixed integer bit-width, it significantly reduces the complexity of MAC operations [37].

## 3. Background

### 3.1. Convolutional Neural Networks

A convolutional neural network (CNN) is a type of deep neural network that processes input images by sliding filters (or kernels) over the data. The CNN structure was developed according to the visual cortex of the animals [38]. CNN models include three key layers for image classification: convolution, pooling, and fully connected layers. The convolution and pooling layers are in charge of extracting and reshaping data features, while the fully connected layer classifies the input data. As the name of the deep learning model implies, the convolution layer plays a critical role in all CNN models.

**Figure 1:**
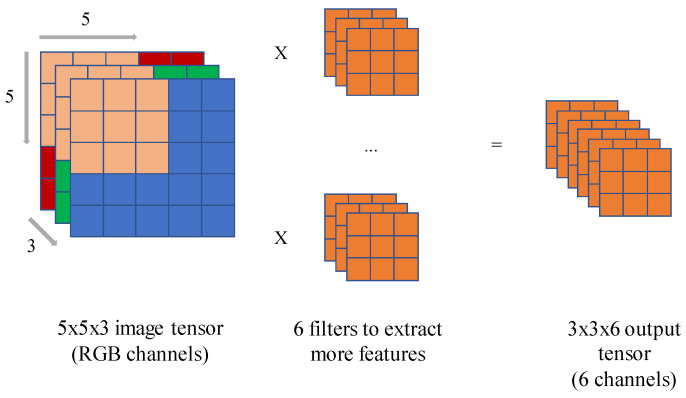
Using multiple filters with three kernels on RGB image tensor.

The convolution layer reduces the input data (i.e., image) into a specific form called a feature map that is much easier to process while learning and preserving the features of the data and passing them to the next layer. The computing system represents an image as a grid of pixels with values ranging from 0 to 255, representing the pixels’ intensity as shown in Figure 1. Since an image usually has multiple channels to represent the color, it is viewed as a tensor, a multidimensional extension of a matrix in the convolutional layer. To extract feature maps, the convolution layer uses filters to perform convolution operations on the tensor. The filter is a fixed-size grid with fixed values inside each cell that slides over the tensor while performing element-wise multiplication for each filter value with a corresponding pixel of the tensor. As shown in Figure 1, the result of this element-wise multiplication is summed up to produce an output tensor called the output feature map. If an image contains multiple channels, a filter is made up of multiple kernels. To extract more features from an input image, multiple filters can be used. The output data from the convolution operations are passed to the nonlinear activation functions, which serve as triggers. There are several types of activation functions such as sigmoid, tangent (tanh), and ReLU (the rectified linear unit).

The convolution layer is followed by a pooling layer that provides a down-sampling operation. The reduction in the image size is a technique to decrease the complexity of the following layers in the convolutional neural networks. Additionally, the pooling layer serves as a noise-suppression layer that helps to strengthen the main features and delete the ones that create noise. There can be multiple types of pooling layers, such as a max pooling and global average pooling layers. The pooling layer uses the same kernel technique. However, instead of performing the convolution operation, it extracts either the maximum value (i.e., max pooling) or global average value (i.e., global average pooling) out of the area where the kernel is currently located.

**Figure 2:**
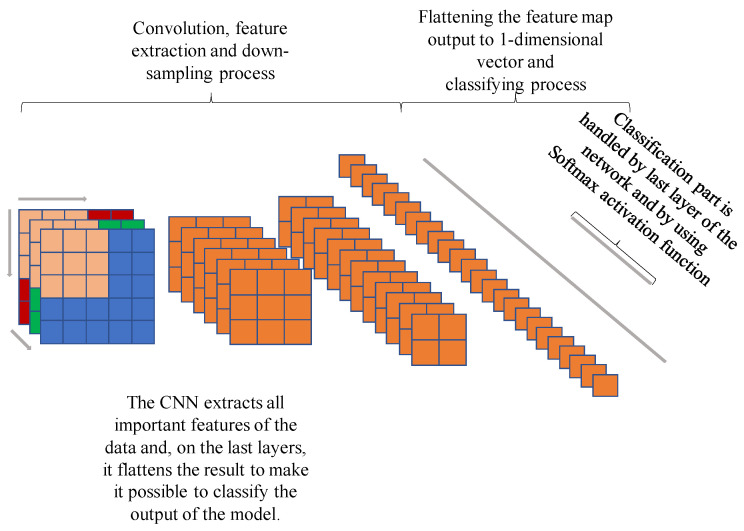
Convolutional neural network (CNN).

The last layers of the convolutional neural networks are fully-connected (FC) layers. The end product of convolution and pooling layers is passed further, serving as input data for the FC layer. To avoid the mismatch of dimensions between the outputs of convolution/pooling layers and the FC layer, the feature maps are flattened before passing them to the FC layer. This flattening operation reshapes the dimensions of feature maps into the vector shape (one-dimensional). The classification procedure is completed by the last layer of the FC layers and, usually, has the same amount of neurons according to the number of classification types.A simple structure of CNN is shown in Figure 2.

### 3.2. Network Quantization

In this subsection, we discuss various post-training quantization techniques such as half-precision floating-point quantization [23,24,28], dynamic range quantization [23,27,28], and full integer quantization [23,26,28].

#### 3.2.1. Quantization Basics

The neural network (NN) model contains neurons with weights, bias, and activation functions. When the neuron receives the input data, it goes through a multiplication procedure with weight value, and the result is passed to the next layer. In a neural network, the weight assigns importance to the input data, influencing the output result.

The multiplication process on a neuron requires the same type of arithmetic operations in accordance with the format of the weights. Hence, this approach increases the computing device’s computation load, which leads to other complications such as energy consumption increase, ambiguous usage of memory, and high latency.

Quantization is a popular model compression technique that reduces the neural network model’s computation load and memory usage by converting the real number values into values with lower precision. As an example, we can consider the conversion of floating-point values into integer values. The quantization function is as follows:

(1) Quant(R)=Int(R/S)−Z,

where *R* is a set of real number values, *S* is a scaling factor, and *Z* is an integer zero point. *Int* maps the output value to an integer range by rounding to the nearest value. This formula represents a uniform mapping of the real values to integer values [28]. The scaling factor *S* is a positive integer that specifies the step size of the quantization. Depending on the scaling factor, the real numbers *R* are partitioned into multiple ranges, and each range of the real number values is mapped to one integer value. The scaling factor *S* is calculated as follows:

(2) $$S=\frac{\beta -\alpha }{2^{b}-1},$$

where [*α*, *β*] represent the clipping range and *b* is the bit width of quantization. Meanwhile, the zero point *Z* ensures that zero is precisely represented. If we use zero-padding for the convolution layers of a CNN model, the real value zero needs to be exactly representable in the quantized form. If the zero point does not correspond to the quantized value, the model would have to use different quantized values for zero, introducing inaccuracies in the results.

#### 3.2.2. Half-Precision Floating-Point Quantization

Half-precision floating-point quantization (FP16 quantization) converts model parameters from single-precision floating-point (FP32) format to half-precision floating-point (FP16) format to reduce the model’s size by two times and decrease the inference latency. One of the advantages of the half-precision floating-point (FP16) format is that it can be executed by using GPU hardware. Due to GPU’s structure, it can execute operations in a single or half-precision floating-point format that can be used in our favor to further reduce the inference latency. In addition, due to the parallel computation format and several hundred cores inside of the GPU, this hardware makes it possible to execute the model faster rather than the CPU.

#### 3.2.3. Full-Integer Quantization

Full-integer quantization converts all model parameter values (weights and activation) to a 8-bit integer format. This quantization method notably decreases the model size and inference latency with a small accuracy degradation. Another advantage of full-integer quantization is that it allows the utilization of a neural processing unit (NPU) hardware. NPU is a hardware accelerator specifically designed for running neural network models. Due to its custom hardware architecture, it is possible to execute neural network models with the lowest latency. As we will see in the next section, it processes the input data at a faster speed than other computing hardware. Nevertheless, converting the model from FP32 format to the full-integer format causes an accuracy degradation due to the way it quantizes the model. Fortunately, even though the model accuracy is dropped, it was within an acceptable range. Furthermore, considering the large reductions in memory size and execution time, the trade-offs are insignificant.

**Figure 3:**
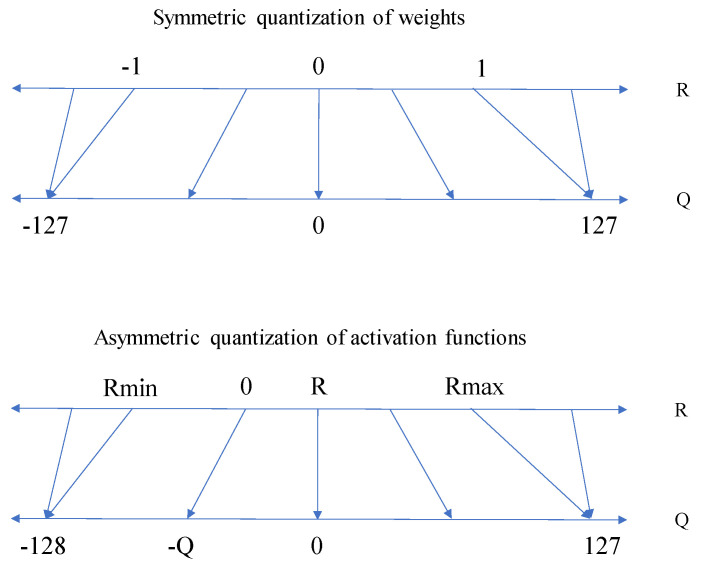
Symmetric quantization of weights (**top**) and asymmetric quantization of activations (**bottom**).

Full-integer quantization quantizes weights as well as activations by scaling them over the range of the 8-bit integer format. It applies symmetric quantization for the weights and asymmetric quantization for the activations. As shown in Figure 3, symmetric quantization is a quantization method that sets the boundaries of parameter values to an equal range (from −1 to 1) and maps them over the range of [−127, 127]. The boundaries of parameter values are called clipping ranges, and their setup procedures are called calibration [28]. If the value is out of range, then the value is clipped to the nearest value. In the case of the asymmetric quantization applied to activation values, the clipping range is not equal (Rmin = −0.5 and Rmax = 1.5). The symmetric quantization is more efficient and less computationally expensive [25] because the zero point value is equal to 0 (*Z* = 0) as shown below:

(3) Quant(R)=Int(R/S),

This is why symmetric quantization is used for weight values. Meanwhile, asymmetric quantization is utilized for activations since applying the range of [−128, 127] to activations provides better accuracy.

#### 3.2.4. Dynamic Range Quantization

**Figure 4:**
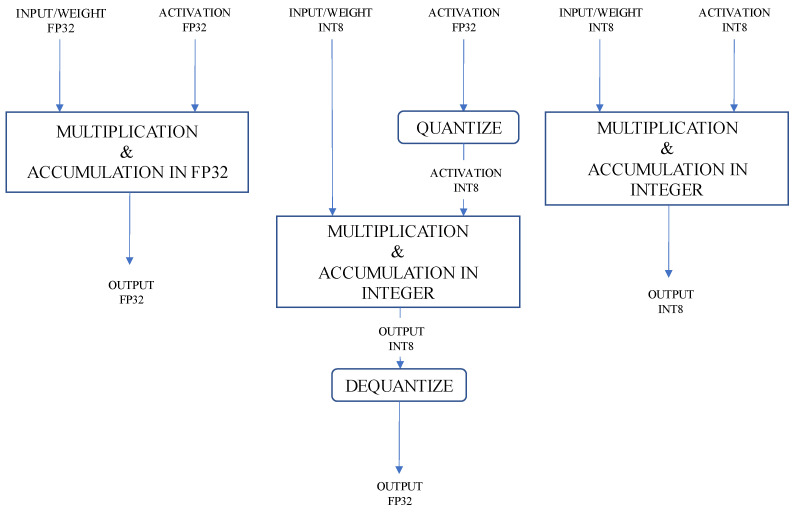
A comparison between full-precision floating-point (**left**), dynamic range quantization (**middle**), and full-integer quantization computation (**right**).

Dynamic range quantization converts all weight values from floating-point precision to integer 8-bit precision. To further reduce the inference latency, “dynamic-range” operators dynamically quantize activations based on their range to 8-bits integer and perform computations with 8-bit integer weights and activations. After multiplication and accumulation, the activation values are dequantized. Figure 4 (middle) shows the overall mechanics of the dynamic range quantization. This optimization provides the inference latency close to the fully fixed-point inference. Overall, performing the multiplication and summation using integer arithmetic provides an accelerated and more efficient execution in terms of execution time and power consumption. Additionally, the model size is reduced even further in comparison to the half-precision floating-point quantization.

The dynamic range quantization symmetrically quantizes the weight values according to the Formula (3) above. On the other hand, the activation value quantization occurs asymmetrically, which is performed according to the Formula (1) above. The dequantization process of activation values is performed according to the Formula (4). The formula below describes how the integer values are dequantized to real values [26]:

(4) R=S(Quant(R)−Z),

where *Quant(R)* is a value from the integer range. From this formula, we can deduce that the real number after dequantization cannot be restored to the same exact value as it was before quantization. Due to this reason, dynamic range quantization may suffer from accuracy degradation. Like full-integer quantization, the dynamic range quantization can reduce the model size and inference time. The difference between full-integer and dynamic range quantizations is that the latter converts the activations to integer format “on-the-fly” during inference time. This is one of the advantages of the dynamic range quantization compared to the full-integer because any representative dataset is not required for the dynamic range quantization. However, since the dynamic range quantization stores the values of activations in a floating-point format during the “stand-by” period, it is impossible to run the quantized model on custom hardware (i.e., NPU) that only supports the low-precision arithmetic operations.

## 4. Convolutional Neural Network for Breast Cancer Diagnosis

### 4.1. Datasets

**Figure 5:**
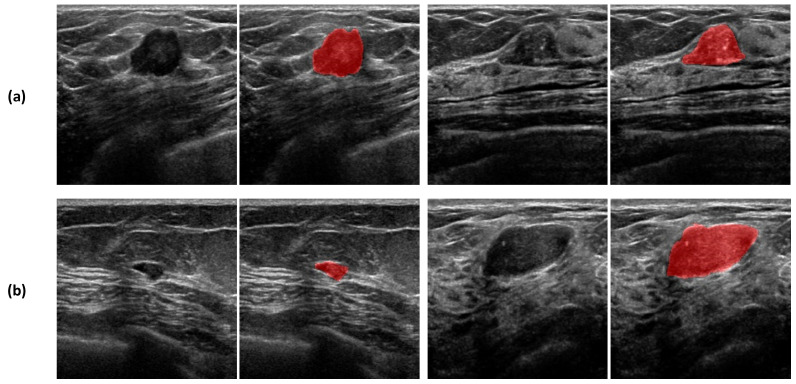
Samples of breast ultrasound images. (**a**) Malignant cases and (**b**) benign cases. Red colored-regions are breast lesions.

In this study, we acquired 1400 ultrasound images of patients with malignant or benign breast tumors from Kyungpook National University Chilgok hospitals. Table 1 is the demographic description of the collected dataset. Senior radiologists with more than 10 years of experience reviewed all acquired images with associated radiological reports. All breast ultrasound images were anonymized by erasing personal information, such as patient name, patient ID, acquisition date, and manufacturer, in the acquired US images using an in-house anonymization software. A sample of ultrasound images are shown in Figure 5.

We randomly divided the acquired data set into training, validation, and test sets with 1000, 200, and 200 images, respectively. The validation set was used to tune the hyperparameters of the classification models. All images were resized to a width of 224 pixels and a height of 224 pixels using bilinear interpolation. The pixel intensities of the images were normalized from 0 to 1 by dividing them by maximum intensity for each image. In the intensity normalization process, we converted the image data type from unsigned int to floating-point (32-bit) to prevent loss of image information by digitization.

### 4.2. Classification Model

**Figure 6:**
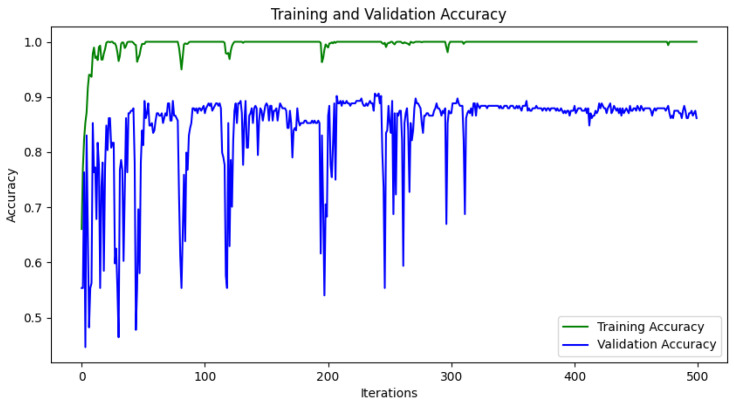
Accuracy per training iteration (Epoch) of VGG16 model.

In this study, we implemented CNN models using VGG16 [39], ResNet34 [40], and GoogLeNet [41] as the backbone networks. The VGG16 is a feedforward CNN with five convolutional blocks. Each convolution block consists of two 3×3 convolutional layers followed by a ReLU activation function and a max-pooling layer. For the classification task, the VGG16 includes fully-connected layers followed by a softmax activation function. However, in this study, we replaced the fully-connected layers with a global average pooling (GAP) layer [42] and a fully-connected layer to acquire class activation maps for each class. We also removed the last max pooling layer of VGG16 to increase the resolution of the class activation maps. Through these modifications, we improved the localization ability of the classifier. Last, we added batch normalization to each convolutional layer in VGG16 for early convergence and robust training against internal covariate shift problem. As shown in Figure 6, the improved CNN model achieved the validation accuracy of 87% at 320 iterations. We also employed the ResNet34 and GoogLeNet by changing their last fully-connected layers for the binary classification of malignancy and benign. We used the same optimizer for the two models as the VGG16 and tuned hyperparameters for both networks using the validation dataset.

## 5. Quantized CNNs for Breast Cancer Diagnosis

**Figure 7:**
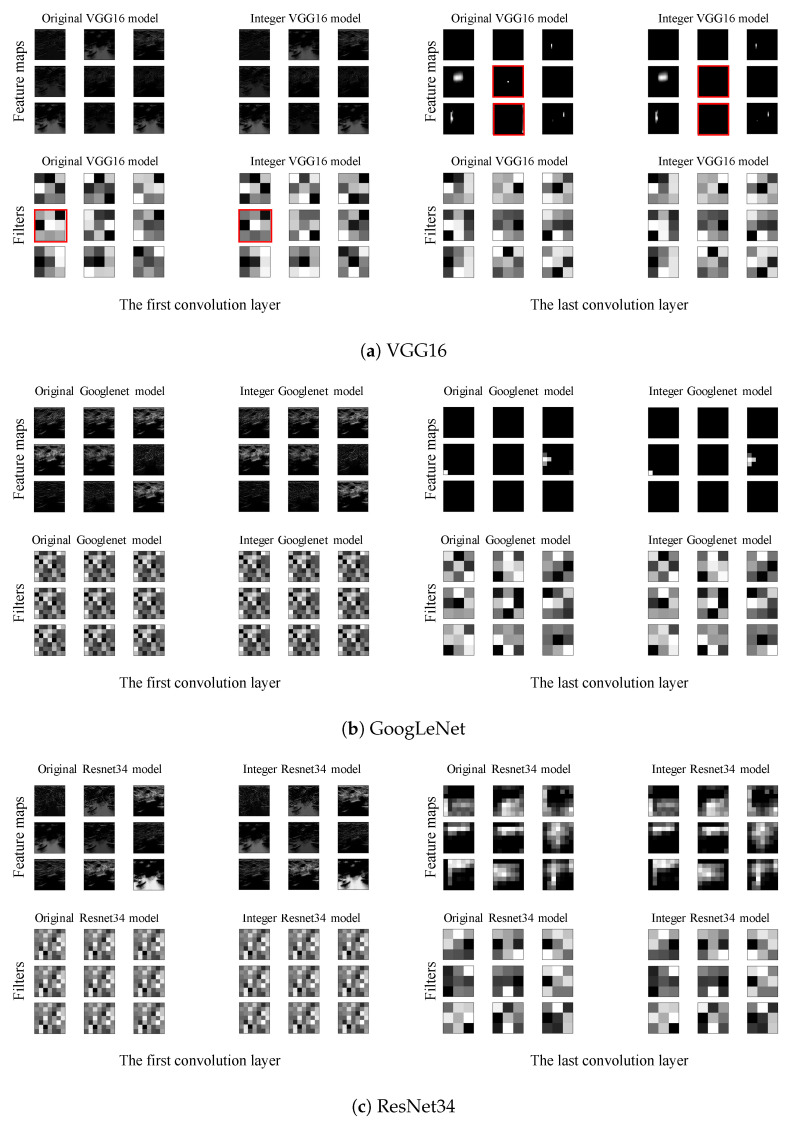
Feature map and filters of the first and the last convolution layers.

In this section, we analyze how the quantization techniques affect the behavior of CNN inference. For this purpose, we observe feature maps and filters of three CNN models specifically implemented using VGG16, GoogLeNet, and ResNet34 networks for the breast cancer diagnosis. Figure 7 shows feature maps and corresponding filters of the first and the last convolution layers of the original and the quantized models. For the quantized models, we applied the full-integer quantization technique. The left part of Figure 7a shows the output feature maps (top) and filters (bottom) of the first convolution layer of the VGG16 models. As shown in figure, there are no significant differences between the feature maps of the original model and those of the quantized model. In the case of filters, some filters in the quantized model have slightly different weight values from the original model, as highlighted with a red box in Figure 7a (left). However, the structure of most filters does not change after the quantization. Similar pattern can be observed in ResNet and GoogLeNet as well. There are no significant differences in the feature maps and the filters between the original and quantized models as shown in Figure 7b,c. Assuming that the structure of the filters remains identical before and after quantization, they extract similar features from the input data, resulting in a trivial accuracy loss after quantization.

Figure 7 also shows the output feature maps and the corresponding filters of the last convolution layers in the original and the quantized models. As shown in figure, the differences in the feature maps and the filters of the last layer between original and quantized models are trivial for all CNN models (VGG16, Googlenet and ResNet34). Since most filters of the quantized models are almost identical to those of the original models, the impact of the quantization on the inference accuracy is not significant, as we will show in Section 8.

**Figure 8:**
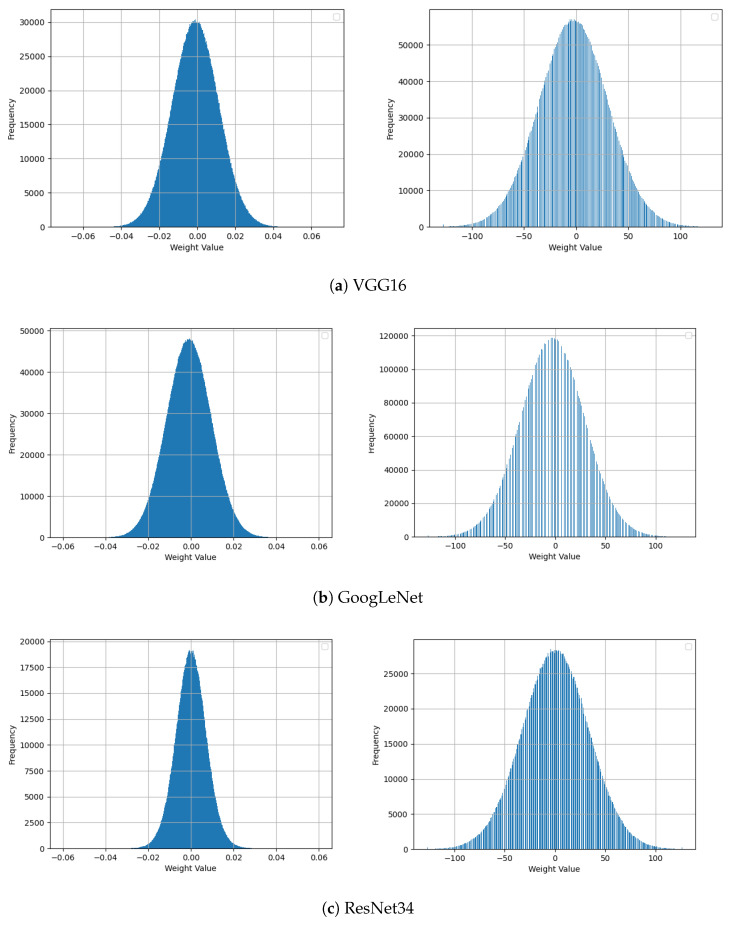
Weight distribution of the last convolution layers of original (**left block**) and integer models (**right block**).

The distribution of weight values in the original and quantized VGG16, GoogLeNet, and ResNet34 models are shown in Figure 8. We can observe that the weight distributions of each model sharply change and shape themselves into an acceptable range of weight values. As we can see in figures, the weight distributions of the original models are denser than those of the full integer models. This is because the original models’ weight values are in single-precision floating-point format, while weights of the quantized models are in integer format. The frequency of the weight values in the full integer model increases due to the reassignment of float-precision values onto a limited range of integer values. Even if the density of weight values is different between the quantized and original models, overall distribution is similar, resulting in the similar filter structures shown in Figure 7.

**Figure 9:**
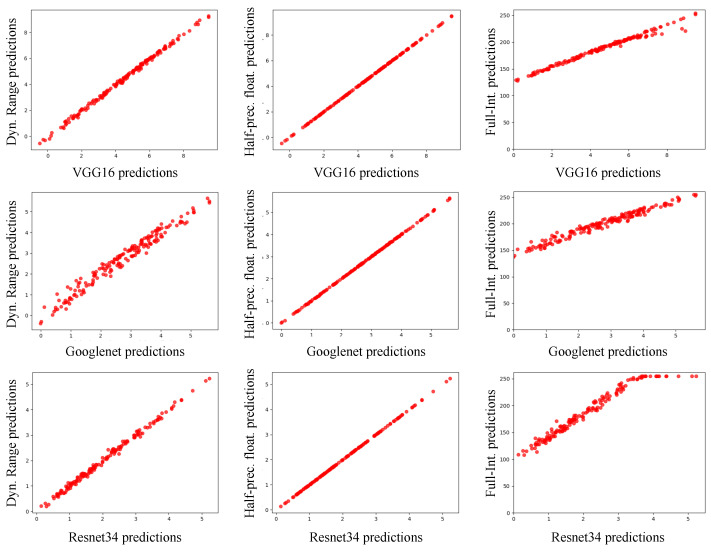
Point cloud graphs for comparing the outputs of original and quantized VGG16, GoogleNet, and ResNet34 models.

We compare the outputs of the original and quantized models for all test images to see if the quantized model produces similar results to the original model. Figure 9 depicts point cloud graphs that compare the outputs of the original model and optimized model quantized using one of three quantization methods. The X-axis represents the original model’s output value, while the Y-axis represents the quantized model’s output value. A red dot in a graph represents a correct prediction for a test image. The CNN model’s output values can be interpreted as confidence in the predicted result. Therefore, red dots near the [0, 0] point represent inference results where the model was less confident in classifying the test images. On the other hand, the red dots in the right and top area of the [4, 4] point represent inference results where the model classifies the test images with greater confidence.

As shown in Figure 9 (middle), the outputs of half-precision models are nearly identical to those of the original models for all test images. In the case of dynamic range quantization, the weights and activation values are aggressively quantized to 8-bit integer format from the 32-bit floating-point format, which can result in value accuracy loss. As a result, the quantized model produces dispersed predictions as shown in Figure 9 (left). In the case of the full-integer quantization, the quantized model’s outputs are biased to a higher confidence level than the outputs of the original model as shown in Figure 9 (right). This is mainly due to inaccurate scaling factors. For full-integer quantization, a small subset of training data is used to estimate the range of the activation values, which are dynamically calculated during the inference time. If the subset of data used to determine the scaling factor is not sufficiently representative, the quantization with the scaling factor will generate significant errors in the quantized activations. Due to this, the full-integer quantization reduces the inference accuracy slightly, as we will show in Section 8.1.

## 6. Hardware Acceleration for CNN Inference in Mobile Processors

Nowadays, the demand to execute computationally intensive workloads, such as deep learning on mobile system is increasing. Therefore, many mobile processors adopt heterogeneous computing architecture to meet the computation demands. They include different hardware accelerators such as GPU (graphic processing unit) and NPU (neural processing unit). By using these accelerators for computation, the mobile processors can achieve higher throughput and power efficiency. In this section, we describe heterogeneous computing resources available in the modern mobile processors.

The central processing unit (CPU) is the most essential and basic processor in any computing device. It can execute operations from solving simple arithmetic to complicated matrix computations. Most of the modern mobile processors use ARM cores as CPUs. For high-performance processing, ARM core employs advanced single-instruction–multiple-data (SIMD) execution engine called NEON. The SIMD engine can execute the same arithmetic operation on multiple different data. NEON’s execution pipeline and dedicated register bank shown as 16 128-bit registers and 32 64-bit registers. With this structure, NEON instructions can execute 16 × 8-bit, 8 × 16-bit, 4 × 32-bit, 2 × 64-bit integer operations, and 8 × 16-bit, 4 × 32-bit, 2 × 64-bit floating-point operations, in parallel.

The graphics processing unit (GPU) was initially designed to accelerate graphic rendering processes. However, as modern GPUs have developed to be programmable, it is now possible to use GPUs for accelerating general-purpose computation. Unlike CPUs, which generally contain a small number of complex cores, GPU usually includes hundreds to thousands of cores inside it. Although the performance of a single core is not high, it facilitates faster and more energy-efficient computation by leveraging a highly parallel structure. Mobile GPUs that are integrated in a chip usually concentrate on energy efficiency over performance.

As the computational cost of CNN inference is very high, demand for dedicated hardware has increased in recent years. To meet this demand, many custom processing engines called NPUs have been developed to accelerate deep learning workloads. NPUs outperform CPUs and GPUs in terms of inference time and energy consumption. One of the common techniques used to implement NPUs is the utilization a systolic array. The systolic array is a structure that interconnects multiple processing engines (PE) that perform a simple task. Once data is given, they flow through this array, and operations are executed at each element within the data. By replacing a processing engine with a systolic array and executing operations, NPUs can achieve massive parallel computation with low energy consumption.

## 7. Experimental Setup

### 7.1. Software Platform

To evaluate the effect of the quantization techniques for the CNN models trained with the breast ultrasound images, we used the Tensorflow [43] and Tensorflow Lite frameworks for model quantization and testing. TensorFlow is an open-source software library developed by the Google Brain team for deep neural networks. TensorFlow Lite is focused on mobile and embedded devices. The main purpose of TensorFlow Lite is to enable machine learning inference directly on mobile devices by putting a lot of effort into three main characteristics: small model size, low energy consumption, and low inference latency [43].

### 7.2. Hardware Platform

We used an evaluation board with the Qualcomm Snapdragon 865 processor for the experiment. The Snapdragon 865 processor contains three types of processing cores: Kryo 585 CPU, Adreno 650 GPU, and Hexagon 698 DSP. Kryo 585 CPU is an ARM-based CPU customized for use in the Snapdragon processor. Since it is based on ARM CPU, Kryo CPU includes the NEON SIMD engine. The Hexagon 698 DSP is a type of NPU specifically designed to accelerate deep learning workloads. Since the Hexagon 698 DSP can only accelerate 8-bit arithmetic operations, we evaluated the inference time of the model quantized to 8-bit integer values (i.e., full-integer quantization) in the experiment.

## 8. Results

### 8.1. Inference Accuracy

The inference accuracy of different CNN models optimized with different quantization techniques is summarized in Table 2. The inference accuracy is evaluated for 200 ultrasonic images with benign and malignant breast cancer tumors. As shown in table, the accuracy of the VGG16 original model without any quantization methods applied (No Opt.) is 87%. Meanwhile, GoogLeNet’s and ResNet’s inference accuracy are 88.5% and 77%, respectively.

The VGG16 and ResNet34 models do not experience any degradation in the inference accuracy when it is quantized with the dynamic range quantization (Dynamic-Range) and the half-precision floating-point quantization (Half-Precision). However, in the case of GoogLeNet, the inference accuracy is slightly reduced (0.5%) when the dynamic range quantization is applied to the model. This accuracy degradation is caused by the aggressive quantization of the weights and activations. Since they are quantized to 8-bit integers from the 32-bit floating-point numbers, the value accuracy may suffer, consequently resulting in relatively high mismatches between the outputs of the original and quantized models, as shown in Figure 9.

The inference accuracy is also slightly reduced for all models when using the full-integer quantization (Full-Integer); the inference accuracy is 0.5% less than the original models. The accuracy degradation with the full-integer quantization could be greater than other quantization techniques. This is because the scaling factor is solely determined by the representative input datasets used for the quantization.

Even though the dynamic-range quantization and the full-integer quantization reduce the inference accuracy, the loss in the accuracy is less than 1%, which can be offset by reduced model size and inference latency.

### 8.2. Model Size

After applying the quantization techniques, the size of CNN models is significantly reduced as shown in Table 2. The original model sizes of the VGG16, GoogLeNet, and ResNet34 are 74.1 MB, 61.7 MB, and 85.1 MB, respectively. The half-precision floating-point quantization (FP16) reduces the model sizes by two times, while the inference accuracy is preserved, as we just discussed in Section 8.1. After applying dynamic range and full-integer quantizations, the model sizes are reduced by around four times. The model size of VGG is reduced to about 18.6 MB. The model size of GoogleNet and ResNet models are reduced to 15.6 MB and 21.5 MB, respectively. This significant reduction in the model sizes leads to lower consumption of memory storage, enabling the deployment of the complex CNN models to mobile devices. Dyanmic range and full-integer quantizations result in the same model size because they use the same target format during the quantization. Both quantization methods use full-integer quantization (8-bit integer) for weights and activations. However, the dynamic range quantization dynamically quantizes activations during inference time, as described in Section 3.2.

### 8.3. Inference Time

**Figure 10:**
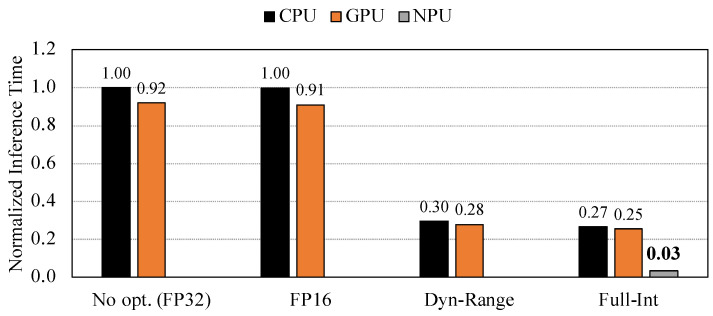
Inference time of the quantized VGG16 models on CPU, GPU, and NPU.

Figure 10 compares the inference time of the VGG16 original and quantized models on each hardware accelerator of the Snapdragon 865 HDK board. All results are normalized to the inference time of the original model on the CPU core. The result demonstrates how the quantization technique can effectively reduce the inference latency in mobile processors. On the CPU core, the dynamic range and the full integer quantizations achieve around 3.8× speed. The GPU core shows slightly better performance than the CPU core. With the GPU, the inference time is reduced by 8% and 9% for the baseline and half-precision floating-point quantizations, respectively, compared to the CPU core.

The half-precision floating-point quantization did not show any improvements in latency, which led us to conclude that the half-precision floating-point quantization can decrease the inference latency only on specific devices which support half-precision floating-point format computation. In our experiment, the FP16 quantization model was automatically up-sampled from FP16 precision to FP32 precision while performing the CNN inference.

**Figure 11:**
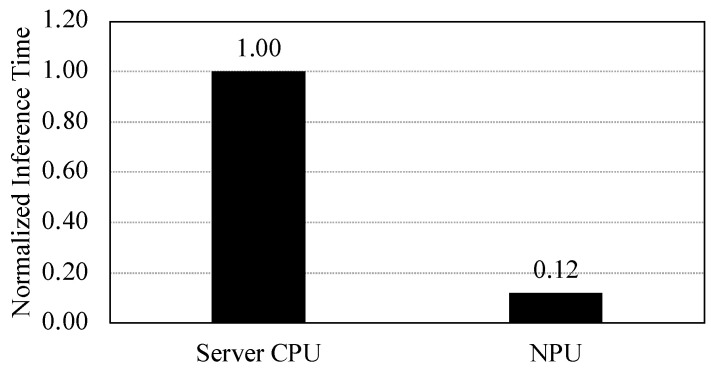
Inference time of the quantized VGG16 models on server-class CPU and NPU.

The NPU core shows a remarkable increase in speed for the model quantized with the full-integer quantization. It achieves about 33× faster inference time than the original model on the CPU core. We also compared the inference time of the CNN models on the NPU with that on the server-class CPU (i.e., Intel Xeon 5215 processor). We evaluated the inference time of the original model for the server-class CPU. As shown in Figure 11, the NPU of the mobile processor is 8.3 times faster than the server-class CPU when it runs the model that is quantized with full-integer quantization technique. This result indicates that mobile processors can outperform server-class processors in terms of the inference latency when the model is appropriately quantized and accelerated on NPU cores specifically designed for the neural network workloads.

## 9. Conclusions

Convolutional neural networks (CNN) have recently received a lot of attention in medical image analysis because they can analyze and classify images faster and more accurately than humans. Using CNNs on portable medical devices allows for quick and accurate disease diagnosis. However, it is difficult to run CNN models on portable medical devices with limited computing resources. This paper demonstrated that the model quantization techniques significantly reduce the model size and computational load of CNN models, enabling the deployment of CNN models on portable medical devices.

## Figures and Tables

**Table 1:** Demographic description of breast cancer US dataset.

	Age	Tumor Size
Benign	44.9±8.8	9.70±5.5
Malignant	51.2±10.4	19.1±9.0

**Table 2:** Inference accuracy and model size.

Opt. Type/Model Type	VGG16		GoogleNet		ResNet34	
	Acc.	Size	Acc.	Size	Acc.	Size
No opt. (FP32)	87.0%	74.1	88.5%	61.7	77.0%	85.1
Dynamic-Range	87.0%	18.6	88.0%	15.6	77.0%	21.5
Half-Precision (FP16)	87.0%	37.0	88.5%	30.9	77.0%	42.6
Full-Integer (INT8)	86.5%	18.6	88.0%	15.6	76.5%	21.5

## Data Availability

Not applicable.
